# The status quo of short videos as a source of health information regarding bowel preparation before colonoscopy

**DOI:** 10.3389/fpubh.2024.1309632

**Published:** 2024-02-13

**Authors:** Foqiang Liao, Yunfeng Huang, Yongkang Lai, Junfeng Xie

**Affiliations:** ^1^Department of Gastroenterology, Jiangxi Medical College, Affiliated Ganzhou People’s Hospital, Nanchang University, Ganzhou, China; ^2^Department of Gastroenterology, The First Affiliated Hospital of Nanchang University, Nanchang, Jiangxi, China

**Keywords:** bowel preparation, colonoscopy, short videos, health information, overall quality

## Abstract

**Background:**

For high-quality colonoscopies, adequate bowel preparation is a prerequisite, closely associated with the diagnostic accuracy and therapeutic safety of colonoscopy. Although popular-science short videos can help people quickly access health information, the overall quality of such short videos as a source of health information regarding bowel preparation before colonoscopy is unclear. Therefore, we intend to conduct a cross-sectional study to investigate the quality of bowel preparation information before colonoscopy through short videos taken on TikTok and Bilibili.

**Methods:**

The Chinese phrases “colonoscopy” and “bowel preparation” were used as keywords to search for and screen the top 100 videos in the comprehensive rankings on TikTok and Bilibili. The Global Quality Score (GQS) and the modified DISCERN score were used to assess the quality of the information provided in these short videos.

**Results:**

A total of 186 short videos were included in this study; 56.5% of them were posted by health professionals, whereas 43.5% of them were posted by nonhealth professionals. The overall quality of these videos was unsatisfactory, with a median DISCERN score of 3 (2–4) and a median GQS of 3 (3–4). The radar maps showed that videos posted by gastroenterologists had higher completeness scores regarding outcomes, management, and risk factors, while nongastroenterologists had higher completeness scores concerning adverse effects, symptoms, and definitions of bowel preparation. Additionally, the median DISCERN score and GQS of the videos posted by gastroenterologists were 3 (3–4) and 3 (3–4), respectively, whereas the quality of the videos posted by patients was the worst, with a median DISCERN score of 2 (1–2) and a median GQS of 2 (1.25–3).

**Conclusion:**

In conclusion, the overall quality of health information-related videos on bowel preparation before colonoscopy posted on specified short video platforms was not satisfactory. Gastroenterologists provide more information on the outcomes, management, and risk factors for bowel preparation before colonoscopy, while nongastroenterologists focus on adverse effects, symptoms, and definitions of bowel preparation.

## Introduction

Colonoscopy is an important tool for the early diagnosis of colorectal diseases, screening for colorectal cancer, and early treatment of colorectal diseases, and bowel preparation is essential before colonoscopy (1, 2). Adequate bowel preparation is a prerequisite for high-quality colonoscopy and is closely associated with diagnostic accuracy and therapeutic safety. Inadequate bowel preparation can lead to prolonged operating time, increased difficulty of colonoscopy, incomplete examination, risk of lesion leakage, and increased risk of complications (2–4). Therefore, scholars have proposed many solutions to improve bowel preparation quality, including newly designed booklets and telephone re-education (5). These methods improve bowel preparation quality to a certain extent, but their implementation requires abundant human and material resources. In China, where medical resources are relatively scarce, it is difficult to provide additional health guidance to all patients who need to undergo colonoscopy; therefore, more convenient and effective health education measures are urgently needed.

Social media is influencing people’s daily lives in unprecedented ways. A total of 4.88 billion people use social media, accounting for 60.6% of the global population. The diversified development of social networking platforms, such as TikTok and Bilibili, has promoted the rapid development of public health information dissemination in China as well as across the world (6). Some studies have investigated health-related information provided on social media platforms, such as dietary information for inflammatory bowel disease (IBD), kidney diseases, and cancer (7–9). One such essential topic is inadequate bowel preparation, which is a major barrier to colonoscopy (2). In this era of social media, encouraging and properly guiding social media users to spread high-quality information on bowel preparation can help people aware of the need for and importance of adequate bowel preparation. Tiktok and Bilibili are two of the most popular social media platforms in China, with hundreds of millions of daily active users (10, 11). Short video sharing platforms disseminate information in the form of vivid animation, and ordinary people can also quickly obtain the health information they want to know by searching keywords on the platform. Therefore, more and more people tend to seek health help on social media platforms. However, the quality of relevant information on the internet varies greatly, and for a large part of the patient population, online information is complicated to understand (12). Additionally, many medicine- and/or science-related videos are posted by laypeople with no medical specialty training, which leads to confusing and/or inaccurate or biased information that may mislead patients and even negatively affect their health. Previous studies have evaluated the quality of health information for several diseases on Tiktok and Bilibili (13–15), but the overall quality of short videos on health information for bowel preparation before colonoscopy is unclear. Therefore, we intend to conduct a cross-sectional study to investigate the quality of bowel preparation information before colonoscopy through short videos taken on TikTok and Bilibili.

## Materials and methods

### Data collection

All the collected videos were extracted from two Chinese short-video platforms (TikTok and Bilibili). From August 15 to August 17, 2023, the Chinese terms “colonoscopy” and “bowel preparation” were used as keywords to search and screen the top 100 videos in the comprehensive ranking on TikTok and Bilibili. The exclusion criteria were as follows: (1) duplicated videos; (2) irrelevant to the topic; (3) videos with no sound or poor sound quality; (4) advertisements; and (5) videos not in Chinese. Fourteen videos were excluded, and 186 videos were included in this study. The content of each video was evaluated by two independent investigators (Liao F and Huang Y), and the following basic information was recorded: publication date, the name and identity authentication of the uploader, titles and departments of health professionals, video content, video duration, and the number of likes, collections, and shares.

### Evaluation methodologies and procedure

Before screening the videos, the guidelines for bowel preparation before colonoscopy, the Global Quality Score (GQS), and the modified DISCERN tool were reviewed and discussed in detail by two investigators. The GQS is a widely used tool for assessing video quality. It is divided into five levels according to the health information quality rating, from very poor to very good, and is registered as a score ranging from 1–5 (16, 17). The modified DISCERN is a widely accepted tool that rates the reliability of a video based on the following five parameters: clarity, relevance, traceability, robustness, and fairness (18, 19). The investigators assessed the video content on the basis of whether it adhered to the above five parameters and calculated a cumulative DISCERN score, which ranged from 0 to 5 points. The comprehensiveness of the video content was evaluated according to the following aspects: whether the videos mentioned definitions, symptoms, risk factors, management, outcomes, or adverse events. Based on whether the video content met the above six criteria and whether the discussion was sufficient, the videos were divided into three categories as follows: no content (0 points), some content (1 point), and extensive content (2 points). Additionally, whether the videos mentioned dietary recommendations for bowel preparation, recommended bowel preparation drugs, or provided medication instructions was recorded. In the health professional group, people who were officially certified as practicing physicians or nurses were included regardless of their department. The nonhealth professional group mainly included nonprofit organizations, science bloggers, and patients.

### Statistical analysis

SPSS version 26.0 (IBM; Chicago, IL, United States) and R statistical software version 4.2.3^1^ were used for statistical analysis. All categorical variables are reported as frequencies and percentages, and chi-square tests or Fisher’s exact tests were performed. Continuous variables are presented as the mean ± standard deviation for normally distributed data, and Student’s *t* test was performed to analyze the data. Conversely, continuous variables are expressed as medians and interquartile ranges (IQRs) for abnormally distributed data, and the Mann–Whitney U test was used to analyze the data. A two-tailed *p* < 0.05 was considered to indicate statistical significance.

## Results

### Overview of the video characteristics

**Figure 1:**
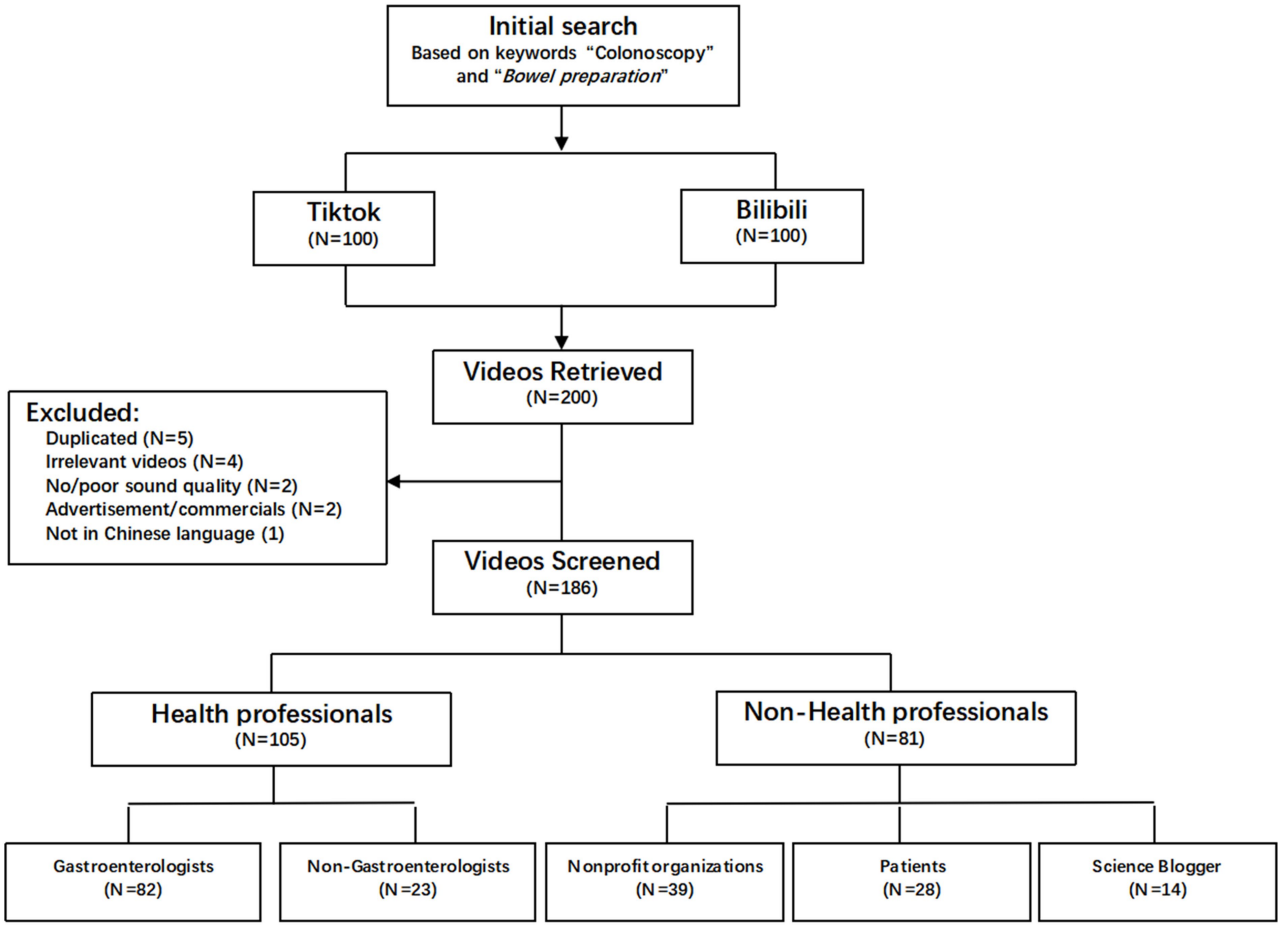
The flow chart of the included videos.

**Figure 2:**
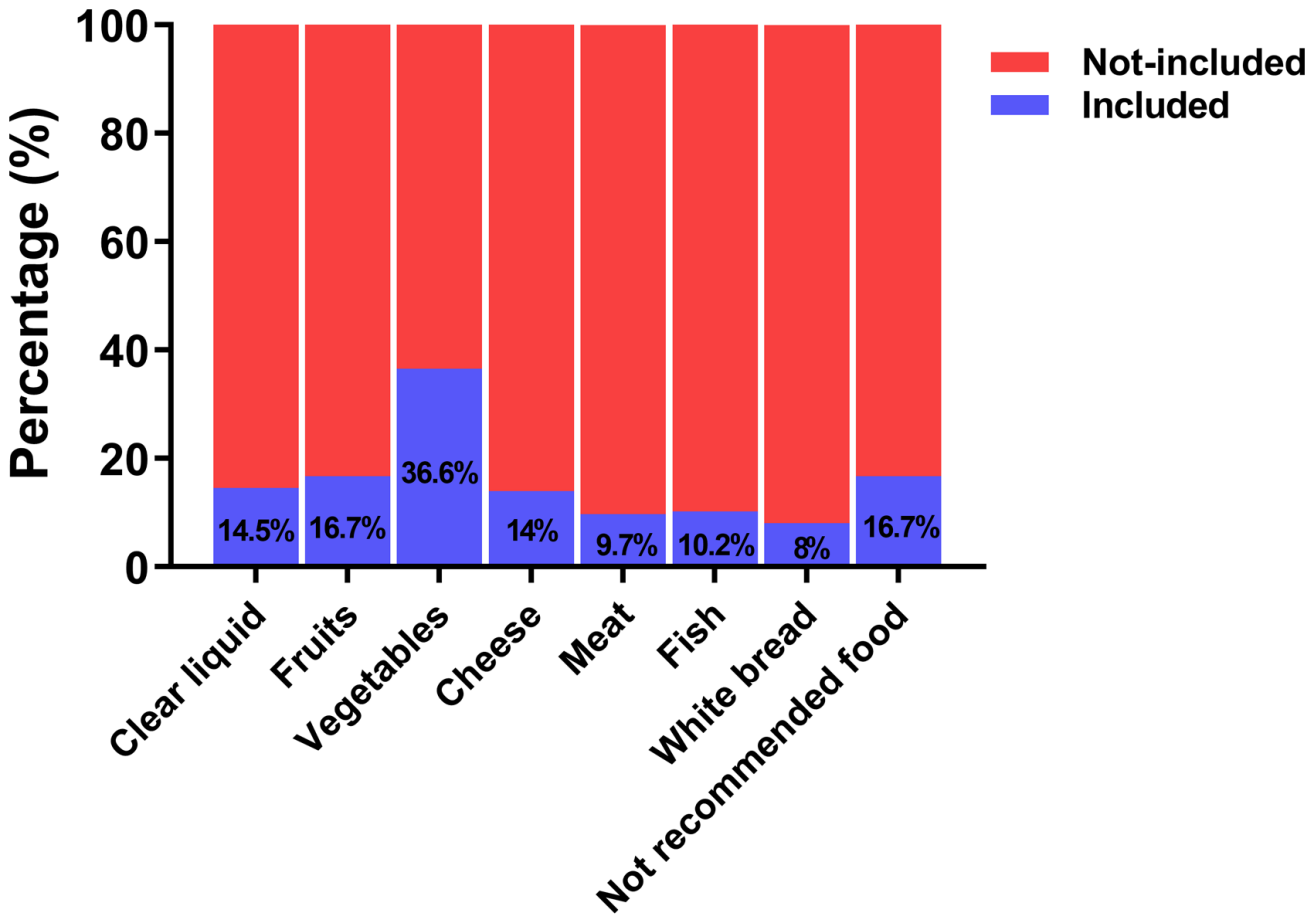
The dietary recommendations are mentioned in a short video.

The top 100 videos in the comprehensive ranking on TikTok and Bilibili were screened, and a total of 186 short videos were eventually included in this study (Figure 1). A total of 56.5% of the videos were posted by health professionals, and the remaining 43.5% were uploaded by nonhealth professionals (Table 1). Among them, gastroenterologists posted the most videos (44.1%), followed by nonprofit organizations (21.0%), patients (15.0%), nongastroenterologists (12.4%) and science bloggers (7.5%). The median duration of the included videos was 96 (IQR: 57–234) seconds. The median number of likes received was 86 (9–448), and the median numbers of collections and shares were 18 (3–136) and 28 (5–147), respectively. The overall quality of these videos was unsatisfactory, with a median DISCERN score of 3 (2–4) and a median GQS of 3 (3–4). A total of 54.8% of the videos provided dietary recommendations during bowel preparation, 46.2% recommended bowel preparation medications, and 36% explained how to consume these medications (Table 2). Figure 2 depicts the dietary recommendations provided in the short videos; the most commonly recommended foods were vegetables and fruits. Additionally, 16.7% of the videos provided some unique food recommendations.

**Table 1:** Characteristics of the sources of bowel preparation–related videos.

Source	Description	Tiktok (*n* = 92)	Bilibili (*n* = 94)	Total (*n* = 186)
Health professionals				
Gastroenterologists	Certified physician or nurse practitioner specializing in gastroenterology	61 (66.3)	21 (22.4)	82 (44.1)
Nongastroenterologists	Certified physician or nurse practitioner specializing in in other medical fields except gastroenterology	14 (15.2)	9 (9.6)	23 (12.4)
**Overall**		75 (81.5)	30 (32.0)	105 (56.5)
Non-health professionals				
Nonprofit organizations	Organizations and public hospitals that operate in the collective, public or social interest	14 (15.2)	25 (26.6)	39 (21.0)
Science blogger	Individuals engaged in the dissemination of scientific knowledge	1 (1.1)	13(13.8)	14 (7.5)
Patients	Patients who have undergone *H. pylori* testing or treatment	2 (2.2)	26 (27.6)	28 (15.0)
**Overall**		17 (18.5)	30 (68.0)	81 (43.5)

**Table 2:** Characteristics of bowel preparation-related videos.

Characteristics	*N* = 186
Video duration (seconds), median, IQR	96 (57–234)
Number of likes, median, IQR	86 (9–448)
Number of collections, median, IQR	18 (3–136)
Number of shares, median, IQR	28 (5–147)
DISCERN score, median, IQR	3 (2–4)
GQS, median, IQR	3 (3–4)
Dietary recommendations (*n*, %)	
Not mentioned	84 (45.2)
Mentioned	102 (54.8)
Recommendations for bowel preparation drugs (*n*, %)	
Not mentioned	100 (53.8)
Mentioned	86 (46.2)
Medication Instructions (*n*, %)	
Not mentioned	119 (64.0)
Mentioned	67 (36.0)

### Video content

**Figure 3:**
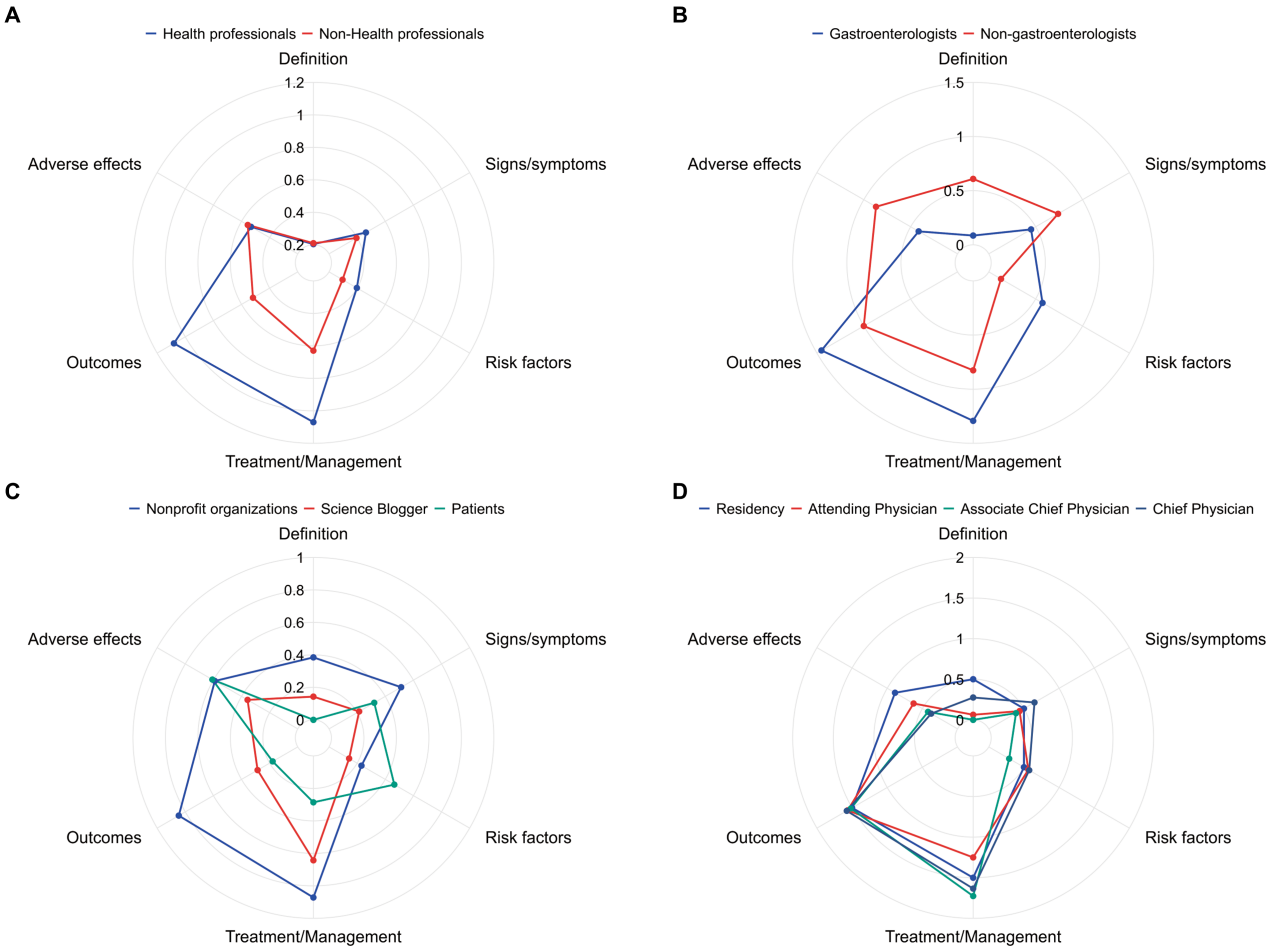
Comparisons of content comprehensiveness from different sources. **(A)** Health professionals and nonhealth professionals; **(B)** gastroenterologists and nongastroenterologists; **(C)** nonprofit organizations, science bloggers, and patients; **(D)** residents, attending physicians, associate chief physicians, and chief physicians.

In terms of the comprehensiveness of each video, the most common topic was bowel preparation management, with 64% of the videos providing relevant information. A total of 62.9% of the videos described the outcomes of bowel preparation, which helps people understand information related to bowel preparation quality. However, topics such as the definition, symptoms, risk factors, and adverse effects were less discussed (Table 3). Furthermore, we evaluated the completeness of the short videos from different sources. As shown in Figure 3A, the videos posted by health professionals showed better content comprehensiveness than did the videos posted by nonhealth professionals. Furthermore, the former provided significantly more information about bowel preparation management and outcomes. Among health professionals, the videos posted by gastroenterologists had higher completeness scores regarding outcomes, management, and risk factors, while nongastroenterologists had higher completeness scores concerning adverse effects, symptoms, and definitions of bowel preparation (Figure 3B). In the nonhealth professional group, nonprofit organizations provided significantly more comprehensive content than did science bloggers and patients (Figure 3C). Figure 3D shows the video content provided by doctors with different professional titles. The results showed that chief physicians focused on bowel preparation management, whereas residency physicians focused on adverse effects and definitions.

**Table 3:** Completeness of video content.

Video content	Definition, *n* (%)	Signs/symptoms, *n* (%)	Risk factors, *n* (%)	Management, *n* (%)	Outcomes, *n* (%)	Adverse effects, *n* (%)
No content (0 points)	157 (84.4)	114 (61.3)	121 (65.1)	67 (36.0)	69 (37.1)	100 (53.8)
Some content (1 point)	20 (10.8)	58 (31.2)	56 (30.1)	62 (33.3)	50 (26.9)	73 (39.2)
Extensive content (2 points)	9 (4.8)	14 (7.5)	9 (4.8)	57 (30.7)	67 (36.0)	13 (7.0)

### Comparison of different video sources

**Figure 4:**
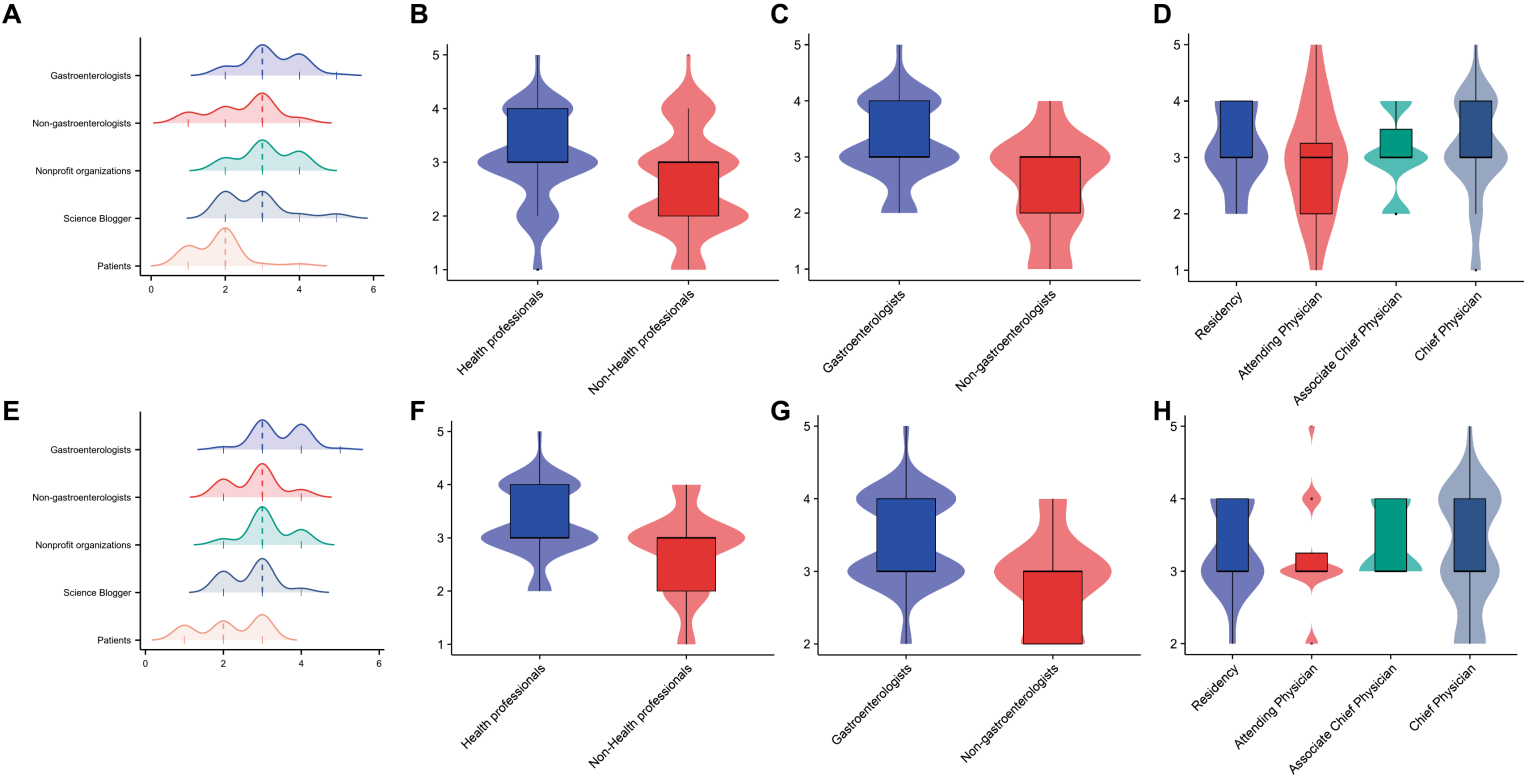
Comparison of DISCERN scores and Global Quality Scores (GQS) for videos from different sources. **(A)** The distribution of DISCERN scores among different sources is displayed in the ridge plot. **(B–D)** Comparisons of DISCERN scores in videos from different sources displayed by violin plots. **(E)** The distribution of GQSs among different sources is displayed by the ridge plot. **(F–H)** Comparisons of GQSs in videos from different sources displayed by violin plots.

Table 4 presents the characteristics and qualities of the videos from different sources. The median duration of videos posted by patients was 350 (219–736) seconds, which was significantly longer than that of videos posted by nonprofit organizations, science bloggers, or health professionals. Although health professionals posted videos for the shortest duration, they were more popular among consumers. The median number of likes, collections, and shares of videos posted by health professionals were 165 (55–803), 37 (5–194), and 51 (8–258), respectively. However, no significant difference was observed in the popularity of the videos posted by gastroenterologists or nongastroenterologists (Table 5). As shown in Figure 4, the quality of the videos posted by health professionals was significantly better than that of the videos posted by nonhealth professionals. The median DISCERN score and GQS of the videos posted by gastroenterologists were 3 (3–4) and 3 (3–4), respectively, which were significantly greater than those of the videos posted by nongastroenterologists. Additionally, videos posted by chief physicians were of better quality. Among nonhealth professionals, the quality of videos uploaded by nonprofit organizations was relatively better, whereas the quality of videos uploaded by patients was the worst, with a median DISCERN score of 2 (1–2) and a median GQS of 2 (1.25–3).

**Table 4:** Comparison of bowel preparation-related video characteristics between health professionals and nonhealth professionals.

Characteristics	Health professionals (*n* = 105)	Nonprofit organizations (*n* = 39)	Patients (*n* = 28)	Science bloggers (*n* = 14)	*p*
Video duration (seconds), median, IQR	69 (51–136)	122 (71–237)	350 (219–736)	94 (58–135)	<0.001
Number of likes, median, IQR	165 (55–803)	15 (3–75)	95 (10–588)	3 (1–16)	<0.001
Number of collections, median, IQR	37 (5–194)	13 (2–38)	10 (0–68)	4 (1–9)	0.001
Number of shares, median, IQR	51 (8–258)	9 (2–38)	36 (2–154)	3 (0–18)	<0.001
DISCERN score, median, IQR	3 (3–4)	3 (3–4)	2 (1–2)	3 (2–3)	<0.001
GQS, median, IQR	3 (3–4)	3 (3–4)	2 (1.25–3)	3 (2–3)	<0.001
Dietary recommendations (*n*, %)					<0.001
Not mentioned	43 (41.0)	12 (30.8)	24 (85.7)	5 (35.7)	
Mentioned	62 (59.0)	27 (69.2)	4 (14.3)	9 (64.3)	
Recommendations for bowel preparation drugs (*n*, %)					<0.001
Not mentioned	72 (68.6)	10 (25.6)	13 (46.4)	5 (35.7)	
Mentioned	33 (31.4)	29 (74.4)	15 (53.6)	9 (64.3)	
Medication Instructions (*n*, %)					<0.001
Not mentioned	80 (76.2)	13 (33.3)	19 (67.9)	7 (50.0)	
Mentioned	25 (23.8)	26 (66.7)	9 (32.1)	7 (50.0)	

**Table 5:** Comparison of bowel preparation-related video characteristics between gastroenterologists and nongastroenterologists.

Characteristics	Gastroenterologists (*n* = 82)	Nongastroenterologists (*n* = 23)	*p*
Video duration (seconds), median, IQR	68 (47–135)	87 (55–201)	0.416
Number of likes, median, IQR	140 (58–749)	213 (28–1,391)	0.868
Number of collections, median, IQR	33 (6–179)	51 (1–399)	0.929
Number of shares, median, IQR	41 (8–248)	72 (5–276)	0.807
DISCERN score, median, IQR	3 (3–4)	3 (2–3)	<0.001
GQS, median, IQR	3 (3–4)	3 (2–3)	<0.001
Dietary recommendations (*n*, %)			0.780
Not mentioned	33 (40.2)	10 (43.5)	
Mentioned	49 (59.8)	13 (56.5)	
Recommendations for bowel preparation drugs (*n*, %)			0.695
Not mentioned	57 (69.5)	15 (65.2)	
Mentioned	25 (30.5)	8 (34.8)	
Medication Instructions (*n*, %)			0.792
Not mentioned	62 (75.6)	20 (78.3)	
Mentioned	20 (24.4)	5 (21.7)	

## Discussion

Colonoscopy is currently the primary method for screening, diagnosing lesions, and treating colorectal cancers in the colorectal region (20). However, colonoscopy may not detect all colorectal lesions, and intraoperative unclear visibility may lead to missed diagnoses of lesions (21). Adequate bowel preparation before colonoscopy is the foundation for effective colonoscopic examination and previous studies have reported that adequate bowel preparation can significantly improve the detection rate of colorectal lesions (22–24). However, up to 20–25% of colonoscopies are performed after inadequate bowel preparation (25, 26). A Spanish clinical trial reported that using a smartphone application to guide patients helped improve the quality of bowel preparation (27). Compared to plain text information, short videos are usually a more comprehensible and impressive way to disseminate information and thus are more popular among the general public (18). Some research reports suggest that conducting health education through online short videos can significantly enhance patients’ awareness of diseases and improve the quality of medical care (28–30). However, for individuals who may lack sufficient discernment of health information, the spread of misinformation on social media platforms can negatively affect public health (31). In this study, we investigated the overall quality of 186 health education videos on bowel preparation before colonoscopy on TikTok and Bilibili.

The overall quality of the included videos was not ideal. Our findings indicated that while the quality of videos uploaded by health professionals is relatively good, nonhealth professionals also contribute to nearly half of the videos posted, which affects the overall quality. However, due to the lack of effective supervision of short video-sharing platforms, popular science content uploaded by users is often widely spread without reviews, and even some users may choose to publish false information to gain attention. Besides, due to the lack of medical education, some videos posted by patients include their personal experiences subjectively rather than presenting objective facts (32). Such videos do not help the public (33), but we found that they are more popular than nonprofit organizations and science bloggers. Therefore, it is crucial to encourage short video-sharing platforms to strengthen the auditability and supervision of popular science videos. In addition, several recent studies have shown that machine learning methods perform well in identifying video quality (34, 35). Therefore, short video-sharing platforms can try to develop a machine learning model to identify the quality of videos to effectively regulate the quality of videos.

Regarding video content, gastroenterologists primarily focus on informing the public how to prepare the intestine and how to judge the quality of bowel preparation, which is especially important for performing a high-quality colonoscopy. A prospective randomized controlled trial reported that telephone re-education on the details of bowel preparation the day before colonoscopy can significantly improve the quality of bowel preparation and the detection rate of polyps (36). However, in many hospitals in China, it is difficult to conduct telephone re-education for all patients the day before a colonoscopy. Therefore, many doctors can use short video-sharing platforms to conduct health education for patients to improve the quality of bowel preparation. Additionally, the choice of diet during bowel preparation can also affect the quality of bowel preparation, and the guidelines recommend consuming fluids, vegetables, and fruits before colonoscopy (37). When we reviewed the videos, we found that health professionals, nonprofit organizations, and science bloggers paid more attention to dietary recommendations, but only a few videos could provide comprehensive advice. Patient-posted videos focused more on adverse events during bowel preparation. This approach can help to inform the public about possible adverse reactions. However, some videos overstate occasional adverse reactions, which may mislead the general public.

Currently, due to the lack of effective supervision on short video sharing platforms, popular science content uploaded by users is often widely disseminated without proper review. Some users may even choose to publish false information to gain attention. Additionally, as patients lack a medical education background, videos posted by them subjectively include personal experiences rather than presenting objective facts. Such videos are not helpful to the public. Therefore, for the future development of short videos related to bowel preparation, we propose the following suggestions: Firstly, short video sharing platforms should be encouraged to strengthen the review and supervision of popular science videos, and platforms should take down inadequate videos after review. Secondly, relevant government departments should invite authoritative experts in gastroenterology to provide detailed explanations of bowel preparation-related knowledge for the creation of accurate health education content. Furthermore, platforms should highlight officially certified authoritative videos for easy discovery by the public in searches, eliminating the need for individuals to judge the content themselves.

This study has several limitations. We evaluated the video quality of the two most commonly used short video-sharing platforms in China, and the video quality of other platforms still warrants further investigation. In addition, the study included videos in Chinese only, and the quality of the videos provided by short video platforms in other languages was unclear. Furthermore, we evaluated only the quality of the top 100 video content items in the comprehensive ranking, which led to a certain deviation in the overall video quality evaluation on the platform.

## Conclusion

Overall, the quality of health information related to bowel preparation before colonoscopy provided on TikTok and Bilibili is not satisfactory. Videos created by healthcare professionals have higher quality than those from medical institutions and scientific blog authors. Among healthcare professionals, gastroenterologists provide more information on the results, management, and risk factors of bowel preparation, while non-gastroenterologists focus on adverse reactions, symptoms, and definitions of bowel preparation. Short videos have become a significant source of health education, and there is a need for strict scrutiny of the release of health-related short videos. Rules for publishing health education short videos on relevant online platforms should be established to safeguard public health and safety.

## Data availability statement

The original contributions presented in the study are included in the article/Supplementary material, further inquiries can be directed to the corresponding authors.

## Author contributions

FL: Data curation, Formal analysis, Investigation, Methodology, Software, Writing – original draft. YH: Conceptualization, Data curation, Formal analysis, Investigation, Writing – original draft. YL: Investigation, Methodology, Project administration, Validation, Writing – review & editing. JX: Investigation, Methodology, Supervision, Validation, Writing – review & editing.

## Footnotes

^1^www.r-project.org
